# Preparation of Superhydrophobic Polymeric Film on Aluminum Plates by Electrochemical Polymerization

**DOI:** 10.3390/molecules14114737

**Published:** 2009-11-19

**Authors:** Fang Wang, Heyi Luo, Qian Wang, Jinggang Wang, Juan Xu

**Affiliations:** College of Science, Northwest Agriculture & Forest University, Xinong Road No.22, Yangling, Shanxi, 712100, China; E-Mail: liurenzu@sohu.com (J.X.)

**Keywords:** aluminum plate, electrochemical polymerization, polymeric film, superhydrophobic

## Abstract

6-(*N*-Allyl-1,1,2,2-tetrahydroperfluorododecyl)amino-1,3,5-triazine-2,4-dithiol monosodium (ATP) was used to prepare polymeric thin films on pure aluminum plates to achieve a superhydrophobic surface. The electrochemical polymerization process of ATP on aluminum plates in NaNO_2_ aqueous solution and the formation of poly(6-(*N*-allyl-1,1,2,2-tetrahydroperfluorododecyl)amino-1,3,5-triazine-2,4-dithiol) (PATP) thin film were studied by means of optical ellipsometry and film weight. The chemical structure of the polymeric film is investigated using FT-IR spectra and X-ray photoelectron spectroscopy (XPS). Contact angle goniometry was applied to measure the contact angles with distilled water drops at ambient temperature. The experimental results indicate that the polymeric film formed on pure aluminum plates exhibits superhydrophobic properties with a distilled water contact angle of 153°. The electrochemical polymerization process is time-saving, inexpensive, environmentally friendly and fairly convenient to carry out. It is expected that this technique will advance the production of superhydrophobic materials with new applications on a large scale. Moreover, this kind of polymeric thin film can be used as a dielectric material due to its insulating features.

## 1. Introduction 

Due to its low cost, light weight, and high mechanical intensity and easy modeling, aluminum and its alloys have been applied in many fields, such as microphotography, semiconductors, electric devices, optical devices and building materials. The building materials include antennas, door and window jambs, roofs and building enclosures, *etc.* If these building materials had superhydrophobic surfaces they could make the water beads roll off the surface completely and wash off contamination very effectively. Therefore, superhydrophobic surfaces, with a water contact angle greater than 150°and sliding angles less than 5°, have attracted considerable interest in fundamental research and potential industrial applications [1,2,3,4,5]. Wettability of metal surfaces is a very important property, governed by both the chemical composition and geometrical microstructures of the surfaces [6,7,8,9,10,11]. In recent years, many methods have been reported for constructing superhydrophobic surfaces. These artificial superhydrophobic surfaces have been fabricated mostly by tailoring surface topography and using techniques such as chemical etching [12,13], phase separation [14], L–B film [15], chemical vapor deposition [16], sol–gel processing [17], electrochemical deposition [18], and so on. However, all these methods are subject to certain limitations, such as tedious fabrication, severe conditions, expensive materials and poor durability, etc. In this paper, aluminum is used as substrate. A superhydrophobic modified aluminum surface is prepared by a very simple, inexpensive, environmentally friendly and time-saving electrochemical polymerization method. The compound used in our experiment is a kind of organic compound containing hydrophobic -CF_2_- group and -CF_3_ terminal groups.

## 2. Results and Discussion 

### 2.1. Polymeric Film Structures

**Figure 1:**
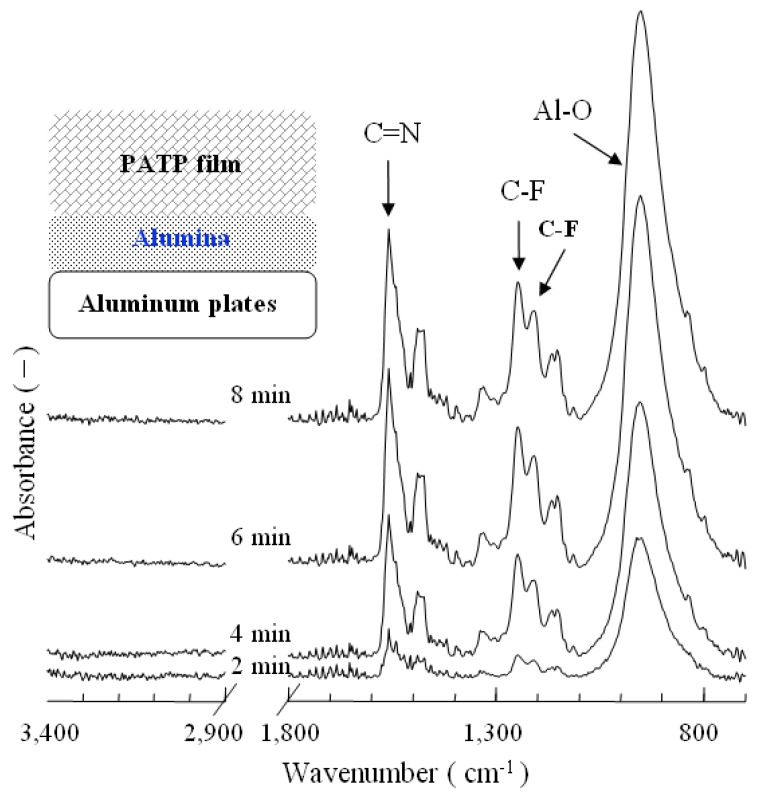
FT-IR spectra of polymeric films obtained by the polymerization of ATP( Resolution: 4 cm^−1^; Incident angle: 80°; Scan times: 128 times).

Electrochemical polymerization on aluminum plates was conducted in 1 mmol/L ATP and 0.15 mol/L NaNO_2_ mixed aqueous solution. To obtain information about the chemical structure of the polymeric film, FT-IR spectra measurements were performed by reflection absorption. Figure 1 shows FT-IR spectra of polymeric films obtained by electrochemical polymerization of ATP on aluminum plates with different polymerization times. In the FT-IR spectrum of aluminum plates coated by polymeric film, the presence of a triazine ring is confirmed by absorption peaks at 1,481, 1,536 and 1,560 cm^−1^, due to >C=N- bonds. Allyl perfluorododecyl amino groups are confirmed by absorption peaks due to C-F stretching vibrations of CF_3_- groups at 1226, 1250 and 1331 cm^−1^ and >CF_2_- groups at 1155 and 1143 cm^−1^. FT-IR spectrum data suggest that the polymeric film on the aluminum plates is composed of poly(6-(*N*-allyl-1,1,2,2-tetrahydroperfluorododecyl)amino-1,3,5-triazine-2,4-disulfide) (PATP). At the same time, the peak of alumina at 956 cm^−1^ is also observed. It suggests that the electrochemical polymerization of ATP and oxidative reaction of aluminum occur simultanously. When the electrochemical polymerization time is over 6minutes, the intensities of >C=N- and C-F absorption peaks appear constant.

In order to determine the optimal electrochemical polymerization time of ATP, the atomic concentrations of ATP films with different polymerization time are investigated by XPS. Table 1 shows the XPS data of PATP films polymerized by different time. It can be seen that the atomic concentrations of C1s, N1s, S2p and F1s on aluminum surface increase with polymerization time. However, these atomic concentrations appear a little diminution with the further polymerization. It is because that there exist the insulating alumina and PATP thin film producing on the aluminum surface within 6 min polymerization time which are blocking the charge transfer process. Therefore, the further PATP films have not been formed any longer even though the polymerization time is prolonged. The results from XPS spectra are consistent with the conclusion from FT-IR spectra.

**Figure 2:**
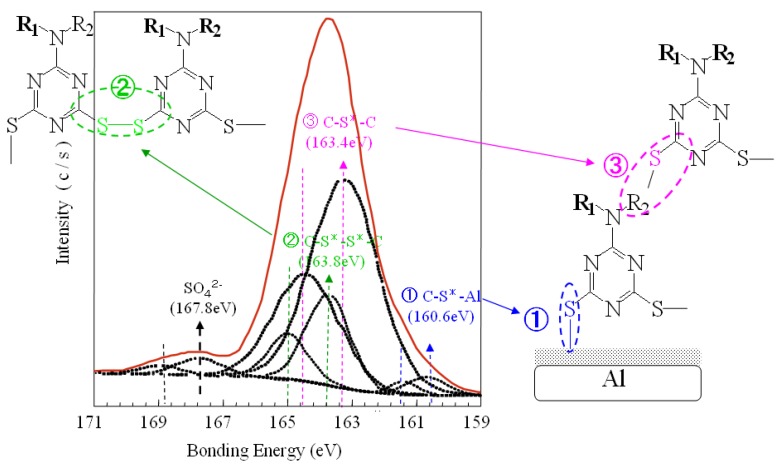
S2p fitted curve of high resolution XPS spectra from polymeric film on aluminum plate in 45 tilt degree (X-ray anode: Al monochromated 2 nm filament; Aperture: 800 × 200 μm).

To confirm the chemical structures of the polymeric films grown on aluminum plates, the S2p fitted curve of XPS spectra was investigated. Figure 2 shows the S2p fitted curve in the XPS spectra of polymeric film on aluminum plate. The S2p spectrum consists of peaks assigned to S*-M (M; aluminum) groups at 160.6 eV, C-S*-C groups at 163.4 eV, C-SS*-C groups at 163.8 eV and SO_4_^2−^ groups at 167.8 eV. Peaks based on S*-M groups indicate the reaction of SH groups with metal during electrochemical polymerization. Peaks based on C-S*-C groups suggest the reaction of SH groups with allylic groups in ATP during electrochemical polymerization. Peaks based on C-SS*-C groups reveal the electrochemical reaction of thiols in ATP during electrochemical polymerization. From the S2p XPS data, the polymeric films generated on aluminum plate are confirmed to consist of poly(6-(*N*-allyl-1,1,2,2-tetrahydroperfluorododecyl)amino-1,3,5-triazine-2,4-disufide). It is assumed that ATP monomer is dissolved as dithiolate anions in the electrolyte aqueous solution and the dithiolate anions transfer two electrons to the anode (aluminum plate) to change to bisthiyl radicals [19].Then the bisthiyl radicals cause coupling with each other to yield PATP film on the aluminum surface.

### 2.2. Properties of Polymeric Film

**Figure 3:**
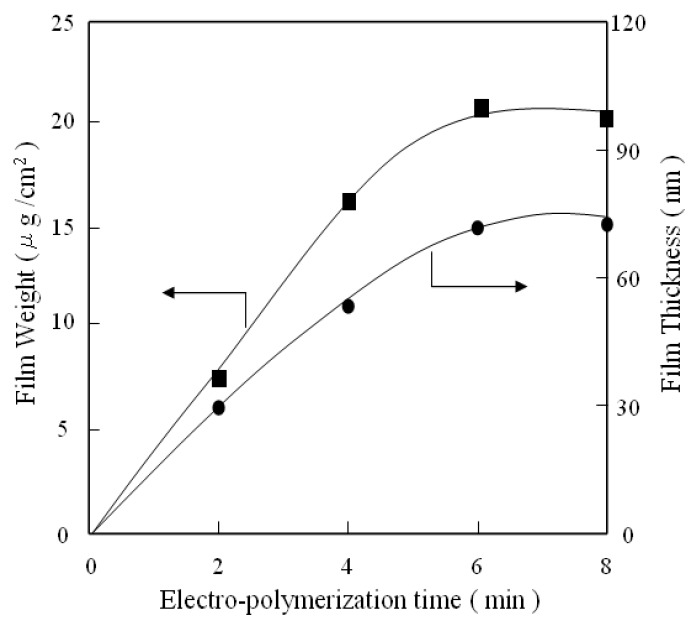
Effect of electrochemical polymerization time on the film weight and film thickness.

The thickness and weight of PATP films on aluminum plates increase with electrochemical polymerization time in proportion to the square root of time, following the parabolic law as shown in Figure 3. Thus, during the PATP films formation process, the diffusion of ATP molecules determine the rate of formation in the same manner as that in the electrochemical polymerization of 6-dioctylamino-1,3,5-triazine-2,4-dithiol monosodium [19]. The diffusion of ATP has great effect on the formation of polymeric films, suggesting that an important factor in electrochemical polymerization is the arrangement of ATP on the metal surface. The polymeric films formed on metal surface are thought to be anisotropic.

**Figure 4:**
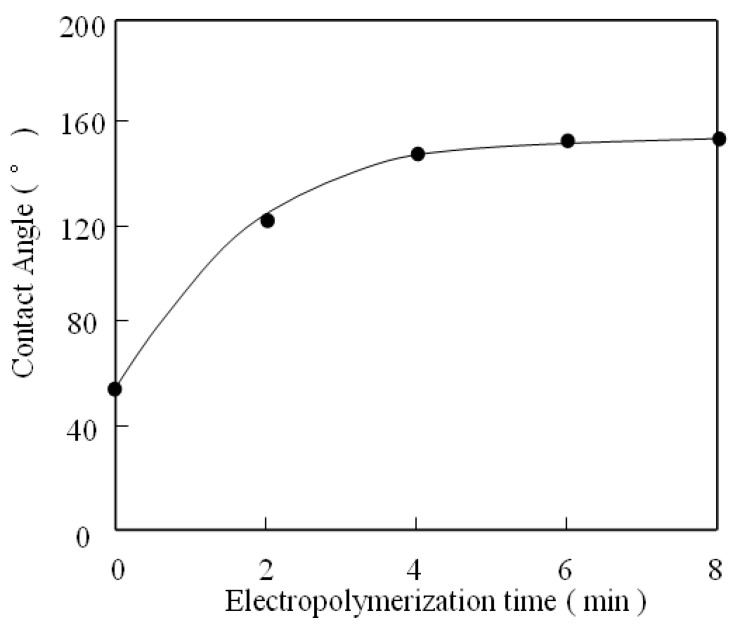
Effect of electrochemical polymerization time on the contact angle.

Figure 4 shows the wettability results for pure aluminum plate and PATP-covered surfaces after different polymerization times. It is obvious that the contact angle of distilled water for a pure aluminum surface without any treatment is 56.3°. 

The contact angle is up to 121.8° after 2 min polymerization of ATP on the aluminum surface. Furthermore, for the aluminum surface covered by more PATP film, the contact angles increase dramatically up to 153°. At the same time, the sliding angle is 3.8° ( the advancing angle is 154° and the receding angle is 151.2°), implying that the water droplets can be moved upward easily even when the surface is only slightly tilted. These results indicate that a superhydrophobic surface is generated on the aluminum plate. The water contact angle and sliding angle did not change after the superhydrophobic surface was exposed to the atmosphere for more than six months. 

According to many research reports, the superhydrophobic property is firmly believed to be due to the presence of binary geometric structures at the micro-/nano-meter scale, and the functional groups in the top layers which reduce the surface free energy [7,8]. In this paper, the aluminum surfaces are covered by heterogeneous PATP films. The CF_3_- terminal group in top layer makes the surface free energy lower. On the other hand, the surface covered by PATP films has the micro- and nano-meter scaled structures, trapping enough air to prevent the penetration of water droplets into the cavities and grooves, which bestow the superhydrophobicity on the surfaces. 

**Figure 5:**
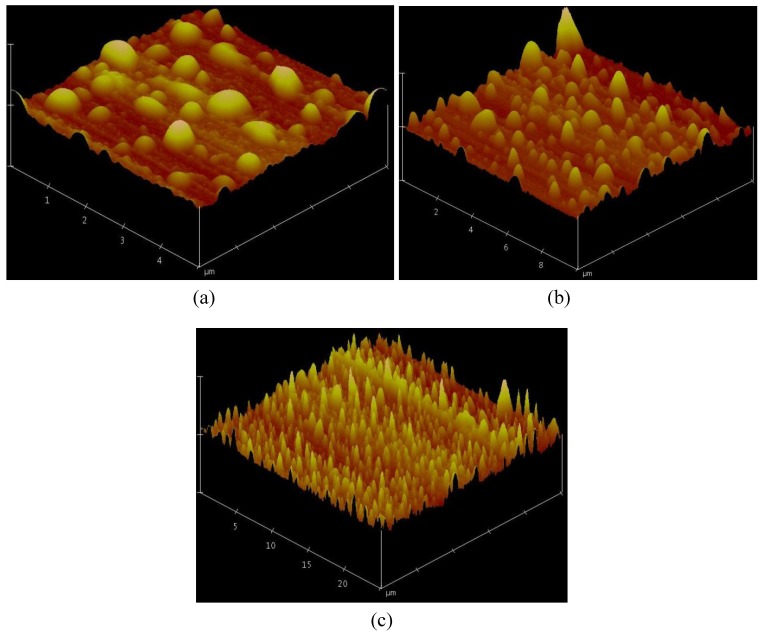
3D AFM images of the aluminum surface covered by PATP films at different scales. (a) 5 × 5 μm; (b) 10 × 10 μm; and (c) 25 × 25 μm.

With the aim of understanding the surface structure, the three dimensional micro-structures formed on the aluminum substrates were observed carefully by AFM. Figure 5 shows the three scales of surface topography observed by AFM. It can be seen that several big mountains, about 5–25 μm, were observed and numerous hillocks were distributed hierarchically around the mountains. With the increasing of electrochemical polymerization time, the data of surface roughness are 9.067 nm, 11.043 nm, 14.251 nm and 18.929 nm. At the same time, the surface roughness before and after the formation of PATPT films was 5.432 nm and 18.929 nm, respectively. These images clearly indicate that the superhydrophobic surfaces of aluminum plates covered by heterogeneous PATP films have binary geometric structures at the micro- and nano-meter scale. According to the above analysis, the micro- and nano-meter structures can bestow the superhydrophobicity on the modified metal surfaces. Actually, the PATP film covered surface can be viewed as having a layer of wax on the aluminum surface. So after the electrochemical polymerization process, the water droplet can not penetrate into the cavities and grooves of the surface as air fills the cavities and grooves. This observation can be explained by Wenzel’s law, which was first derived by Wenzel to describe the CA for a liquid droplet at a rough solid surface [7,20]:

cosθ*_W_* = *r∙*cosθ

where θ*_W_* is the apparent CA in the Wenzel mode, *r* is the surface roughness factor and θ is the CA on the smooth surface made of the same material. From the above equation, it can be found clearly that if the CA of a liquid on a smooth surface is more than 90°, the apparent angle on a rough surface will be larger, while for a true CA less than 90°, the angle on a rough surface will become smaller. In this research, the aluminum surface treated by polymerization of ATP monomer has higher roughness, hence, the CA for the rough surface would increase.

## 3. Experimental 

### 3.1. Materials and Reagents 

Aluminum plates (0.1 × 300 × 300 mm) as a working electrode were purchased from the Nilaco Corporation Company. Aluminum test samples for electrochemical polymerization were prepared by cutting these larger plates into pieces 0.1 × 30 × 50 mm in size. The aluminum plates were degreased by alkaline solution at 60 °C for 10 min, followed by ultrasonic cleaning in acetone and dried in air. ATP monomer with thiol functional groups (-SH) had been synthesized by ourselves [21]. Sodium nitrite, acetone and ethanol were used without any further treatment. Distilled water was used as a solvent. Aqueous NaNO_2_ solution was used as the supporting electrolyte. The concentrations of ATP and NaNO_2_ were 1.0 mmol/L and 0.15 mol/L, respectively.

### 3.2. Electrochemical Polymerization

Electrochemical polymerization of ATP was performed using an electrochemical measurement apparatus (Hotoku Denko Co. LTD HZ-3000) [19,22,23]. The electrolytic cell was equipped with a working electrode (Aluminum Plates, WE), two counter electrodes (Stainless Steel Plates, CE), and reference electrode (Saturated Calomel Electrode, SCE), and filled with an electrolytic solution containing ATP monomer (1 mmol/L) and supporting electrolyte (0.15 mol/L) in distilled water. Electrochemical polymerization of the ATP monomers was performed galvanostatically with 0.30 mA/cm^2^ of current density at 10 °C. After electrochemical polymerization, the working electrode was removed from the electrolyte and immediately rinsed with distilled water, ethanol and then dried in air.

### 3.3. Measurements

Polymeric film thickness was determined using a JASCO M-150i ellipsometer. FT-IR spectra were measured at a resolution of 4cm^−1^ by high-performance reflection absorption spectroscopy using a JASCO IR-5500. A reflection attachment was used at an incident angle of 80° together with a wire grid polarizer. Contact angles of pure water on the treated aluminum plates were determined using an Elma goniometer contact angle measuring apparatus (Elma-type G-1) at ambient temperature. Droplet diameter was controlled at 0.8 to 0.1 mm. X-ray photoelectron spectroscopy (XPS) was performed to determine the elemental composition of the aluminum surface. XPS spectra were obtained using a ULVAC PHI-5600 spectrometer with monochrome Al Kα radiation (1486.6 eV). Samples were examined over an 800 μm area at an incident angle of 45°.

## 4. Conclusions 

In this paper, 6-(*N*-Allyl-1,1,2,2-tetrahydroperfluorododecyl)amino-1,3,5-triazine-2,4-dithiol mono-sodium was polymerized electrochemically on aluminum plate. Chemical structures of the fabricated PATP films were investigated. FT-IR spectra and XPS data of polymeric films suggest that the formation reaction of PATP film and the oxidative reaction of aluminum occurred simultaneously on the aluminum plates. The thickness and weight of polymeric film on the aluminum platea increase in proportion to the square root of polymerization time, following the parabolic law. With an extended polymerization time the thickness and packing density of the polymeric films increase. From the contact angle value (153°), it is assumed that the surface covered by PATP films has micro- and nano-meter scaled structures, and the top layer of the polymeric films consists of CF_3_- groups. It is believed that this method could be easily applied to other metals such as steel, zinc, nickel and so on. Therefore, this study is of great importance for constructing micro- and nano-scale structures to obtain unique properties and it is expected that this technique will advance the production of superhydrophobic materials with new applications on a large scale. Furthermore, this kind of thin film has insulating features [24], so it could be used as a dielectric material.

## Figures and Tables

**Table 1:** Atomic concentrations of polymeric films formed by different electrochemical polymerization time.

Time ( min )	C1s (%)	N1s (%)	S2p (%)	F1s (%)	Al2p (%)	O1s (%)
2	28.11	3.97	1.97	25.97	13.04	26.94
4	35.06	6.72	3.29	44.23	3.81	6.89
6	36.01	7.03	3.45	45.61	2.95	4.96
8	35.58	7.01	3.27	45.76	2.93	5.44
